# Knowledge and attitudes regarding amblyopia among parents in Jeddah, Saudi Arabia: a cross-sectional study

**DOI:** 10.1186/s13104-021-05478-y

**Published:** 2021-02-10

**Authors:** Ahmed Basheikh, Nizar Alhibshi, Motaz Bamakrid, Rasha Baqais, Mohammed Basendwah, Sara Howldar

**Affiliations:** 1 Department of Ophthalmology, Faculty of Medicine, King Abdulaziz University, P.O. Box 80112, Jeddah, 21589 Saudi Arabia; 2 Cardiac Surgery Department, King Fahad Armed Forces Hospital, Jeddah, Saudi Arabia; 3 Faculty of Medicine, King Abdulaziz University, Jeddah, Saudi Arabia

**Keywords:** Amblyopia, Eye care, Knowledge, Parents, Awareness, Screening, Attitudes

## Abstract

**Objectives:**

To assess parents’ perceptions about amblyopia and its causes, symptoms, risk factors, treatments, and the importance of follow-up and to evaluate their awareness regarding the consequences and critical complications.

**Results:**

Parents or their companions (n = 401) were surveyed, and 52.9% were mothers, 81.8% of mothers and 85.7% of fathers were highly educated (diploma, college or university degree), and 58 (14.5%) had a child who was afflicted with amblyopia. The knowledge subscale (nine items) showed acceptable reliability. Twenty percent of the participants declared having adequate knowledge about amblyopia, but assessment by item showed low percentages of an adequate knowledge level, ranging from 0% for amblyopia causes, 35.9% for definition, and 59.9% for whether amblyopia is a hereditary disease. Thus, the rate of adequate knowledge (Knowledge Score ≥ 8) was 25.9%, and was associated with parents’ nationality (p < 0.05) and self-declared knowledge about amblyopia (p < 0.001). No significant difference was observed among respondents who had a child with amblyopia. There was a lack of knowledge about basic information and different aspects of the disease, which emphasized the relevance of school-based screening programs and adequate use of trustworthy internet resources using easily understood medical information.

## Introduction

Amblyopia is defined as a reduction of the best-corrected visual acuity that is caused by abnormal visual development due to impaired visual stimulation [1]. It can be monocular or binocular, without the existence of specific physical or pathological defects [2]. The susceptible age group is between birth and 7 years of age.

Amblyopia is a main cause of visual defects in children. Several studies have shown that amblyopia is the most significant cause of unilateral visual impairment [3, 4]. Recent estimates have indicated that the pooled prevalence of amblyopia worldwide was 1.75% [5]. In Saudi Arabia, different estimates were reported (0.5% among pre-school children in Riyadh, 1.3% among pre-school children in Jeddah, 1.4% in primary school children in Al Hassa, 1.85% among primary school children in Abha, and 3.9% among primary school children in ALQassim) [6–10].

Amblyopia is usually underreported despite the availability of easy diagnostic methods [11]. The inability to diagnose this condition leads to multiple detrimental consequences on contrast sensitivity, visual acuity, and binocular vision. This might interfere with educational attainment and skills, impair social development, and affect future career opportunities [12].

Early diagnosis and treatment are critical to obtain favorable outcomes in patients with amblyopia. Adequate parental knowledge about the disease will help to establish timely consultations and obtain positive outcomes. Lack of knowledge about eye care in the developing countries has led to significant delay between symptom manifestation and clinical presentation [13]. This might be associated with poor compliance to therapy [13, 14]. Even in developed countries, parental knowledge seems to be mediocre [15, 16].

Most of the relevant studies used interviews to collect qualitative data about parental knowledge or relied on a subjective assessment of their familiarity with amblyopia [14–17]. Alternatively, parental knowledge could be assessed via a validated questionnaire to answer more objective knowledge-based questions. Although the questionnaires showed high relevance and readability, none of them were evaluated for test–retest reliability [18]. To the best of our knowledge, there is no available validated questionnaire for this purpose, and little is known about the degree of parental knowledge about ocular diseases in Saudi Arabia. The aim of this study is to assess parents’ perceptions about amblyopia, its causes, symptoms, risk factors, treatment options, and the importance of follow-up, and to evaluate their awareness regarding the consequences and critical complications using a specifically predesigned questionnaire.

## Main text

### Methods

Ethical approval for this cross-sectional study was obtained from the Biomedical Ethics Research Committee at King Abdulaziz University, Jeddah, Saudi Arabia. The study was conducted among parents attending the amblyopia awareness campaign, which took place in the Red Sea Mall, in Jeddah, Saudi Arabia, from 29 to 30 January 2016. The campaign was performed by a group of specialized ophthalmologists and supporting medical students. The campaign’s aim was to raise the awareness about amblyopia among parents and companions to prompt voluntary screening and improve early detection.

All adults accompanied by a child aged 1–16 years of age were approached and invited to participate in the study before any awareness campaign material was provided. The study goals and procedures were explained and individuals who consented were invited to answer a self-administered questionnaire with the assistance of a trained nurse or medical student.

The study used a semi-structured questionnaire, which was divided into four parts: (1) sociodemographic data of the companion and child’s parents; (2) knowledge-related questions consisting of one self-assessed knowledge question “do you have adequate knowledge about amblyopia”, six multiple-choice questions assessing different domains of knowledge about amblyopia including definition, causes, symptoms, risk factors, complications, and management options, and three simple-choice (yes/no) questions including whether the child’s age impact treatment outcome, whether amblyopia requires a lifelong treatment, and whether it is a hereditary disease; (3) attitudes about amblyopia including three key questions: “in your opinion, when is it necessary to take your child for an ophthalmology visit?”, “do you think amblyopia can be cured if the child complies with the treatment”, and “in your opinion, do parents have an essential role in the treatment of amblyopia?” The following three answer options each were given: “yes”, “no”, or “I do not know”; and 4) whether the respondent has a child with amblyopia.

The questionnaire was developed by the authors in collaboration with two consultant ophthalmologists and underwent face and content validity (Additional file 1).

#### Scoring system

To analyze the knowledge levels about amblyopia, a score was calculated based on respondent’s answers to the nine knowledge-related items as follows: (a) *for multiple-choice questions*, the answer was scored 0 if no correct option was given (inadequate knowledge), 1 if at least one correct option was given besides other incorrect option(s) (partial knowledge), and 2 if all correct options were given without any incorrect option (adequate); and (b) *for simple-choice questions (yes or no),* incorrect answers or if respondent replied: “I do not know” were given a score of 0, while correct answers were scored as 1. Thus, a total knowledge score (0–15) was calculated.

#### Statistical methods

Statistical analysis was performed using the Statistical Package for Social Sciences version 21.0 for Windows (SPSS Inc., Chicago, IL, USA). An adequate knowledge level was assumed for any respondent who adequately replied to at least half of the questions, i.e. knowledge score ≥ 8 out of 15. Analysis of reliability of the knowledge subscale (9 items) using the scoring system levels showed a Cronbach’s alpha = 0.748, indicating acceptable reliability. Analysis included a Chi-square test and Fisher’s exact test, as appropriate. A p value < 0.05 was considered to reject the null hypothesis.

### Results

#### Participants’ characteristics

Parents and companions (n = 401) were surveyed, and 52.9% were mothers. Children’s mothers’ characteristics included a relatively young age (mean [standard deviation SD] = 34.45 [5.43] years), high educational level (81.8%), 26.7% were employed, and 53.6% were Saudi citizens. Fathers’ characteristics showed a mean (SD) age = 40.67 (6.61) years, 85.7% were highly educated, 97.3% were employed, and 52.9% were national Saudis. Among all respondents, 58 (14.5%) had a child afflicted with amblyopia (Table 1).

**Table 1:** Participants’ characteristics (N = 401)

Parameter	Category	Frequency	Percentage
Guardian	Mother	212	52.9
	Father	155	38.7
	Other	33	8.2
Mother’s data			
Age	Mean	34.45	–
	SD	5.43	
Educational level	Illiterate	3	0.7
	Primary	20	5.0
	Secondary	43	10.7
	Diploma/college	11	2.7
	University	317	79.1
	Not specified	7	1.7
Profession	Housewife	233	58.1
	Employed	107	26.7
	Retired	1	0.2
	Not specified	60	15.0
Nationality	Saudi	215	53.6
	Non-Saudi	140	34.9
Father’s data			
Age	Mean	40.67	–
	SD	6.61	
Educational level	Illiterate	3	0.7
	Primary	6	1.5
	Secondary	36	9.0
	Diploma/college	19	4.7
	University	325	81.0
	Not specified	4	1.0
Profession	Unemployed	1	0.2
	Employed	390	97.3
	Retired	4	1.0
	Not specified	6	1.5
Nationality	Saudi	212	52.9
	Non-Saudi	145	36.2
Having a child with amblyopia			
	No	343	85.5
	Yes	58	14.5

Because of missing data, some values do not add up to the total

*SD* standard deviation

#### Pattern of answers by knowledge domain

**Fig. 1:**
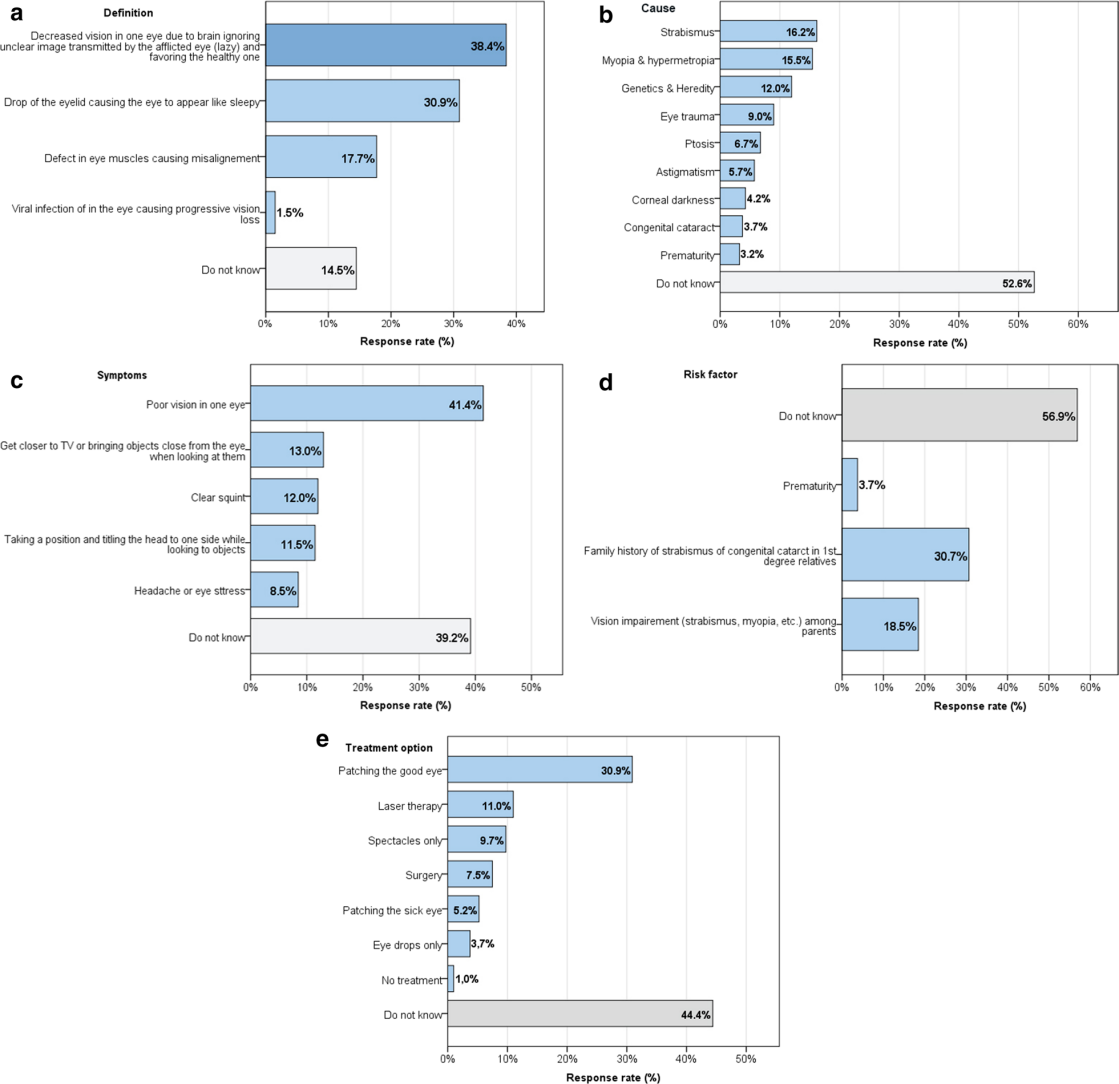
Knowledge about different aspects of amblyopia including **a** definition, **b** causes, **c** symptoms, **d** risk factors, and **e** management. Bars represent the percentage of respondents who replied yes to the given answer option. One respondent may give more than one answer

Figure 1a–e represents the answering pattern to knowledge items including definition, causes, symptoms, risk factors, and management options.

#### Sources of knowledge

Physicians represented the most frequent source of knowledge about amblyopia, as reported by 19.0% of the respondents, followed by the internet (16.0%). Other selected choices included television (7.7%), relatives (7%), newspapers/magazines (4%), and other sources (10.0%).

#### Attitudes regarding amblyopia

To the question “in your opinion, when is it necessary to take your child for an ophthalmology visit?”, 49.1% replied “when there is an abnormal sign”, 30.2% replied “periodic routine check-ups”, and 27.2% replied “when the child complains”. To the question “do you think amblyopia can be cured if the child complies with the treatment”, 55.6% replied “yes”, while 38.7% replied that they did not know. To the question “in your opinion, do parents have an essential role in the treatment of amblyopia?”, 66.6% agreed, while 27.9% replied that they did not know.

#### Assessment of knowledge about amblyopia

Table 2 presents the knowledge levels among the study population for each knowledge-related item. Only 20% of the participants declared having sufficient knowledge about amblyopia. Assessment by item showed that the percentage of adequate knowledge about amblyopia: definition (35.9%), causes (0.0%), symptoms (1.0%), risk factors (5.7%), complications (4.5%), and management (24.4%). For the previous items, the percentage of respondents who gave partially correct answers was 2.5 to 47.1%. Further, 59.4% knew that the child’s age affects the treatment outcome, 59.9% knew that amblyopia is not a hereditary disease, and 39.7% knew that it does not require lifelong treatment.

**Table 2:** Assessment of knowledge about amblyopia and the scoring system (N = 401)

Dimension	Knowledge level (score)	Frequency	Percentage
Self-declared knowledge^1^	Yes	80	20.0
	No	301	75.1
	No answer	20	5.0
Definition	Inadequate (0)	247	61.6
	Partial (1)	10	2.5
	Adequate (2)	144	35.9
Causes	Inadequate (0)	254	63.3
	Partial (1)	147	36.7
	Adequate (2)	0	0.0
Symptoms	Inadequate (0)	211	52.6
	Partial (1)	186	46.4
	Adequate (2)	4	1.0
Risk factors	Inadequate (0)	231	57.6
	Partial (1)	147	36.7
	Adequate (2)	23	5.7
Complications	Inadequate (0)	194	48.4
	Partial (1)	189	47.1
	Adequate (2)	18	4.5
Treatment options	Inadequate (0)	277	69.1
	Partial (1)	26	6.5
	Adequate (2)	98	24.4
Does child age impact the treatment result?	No (0)	36	9.0
	Yes (1)	238	59.4
	I do not know (0)	127	31.7
Does amblyopia require lifelong treatment?	No (1)	159	39.7
	Yes (0)	42	10.5
	I do not know (0)	200	49.9
Is amblyopia a hereditary disease?	No (1)	240	59.9
	Yes (0)	136	33.9
	I do not know (0)	25	6.2

^1^Respondent replied to the question: do you have adequate knowledge about amblyopia?

#### Factors associated with knowledge

Considering the proposed scoring system and for the knowledge score cutoff of ≥ 8/15, the percentage of adequate knowledge among the entire study population was 25.9%. The percentage of adequate knowledge was significantly associated with the mother’s nationality (32.1% among Saudi versus 18.6% among non-Saudi, p < 0.005), father’s nationality (31.1% among Saudi versus 18.6% among non-Saudi, p < 0.008), and self-declared knowledge about amblyopia (51.3% among those who declared having adequate knowledge versus 19.6% among their counterpart, p < 0.001). However, the knowledge level showed no significant association with guardian type, parent’s age or educational level, or whether the respondent had a child with amblyopia (p > 0.05) (Additional file 2).

#### Normality tests

The primary outcome variable, knowledge score, showed mean (SD) = 4.78 (3.26), median = 5.00 (range = 0-13). The variable was not normally distributed, showing Kolmogorov–Smirnov test (statistics = 0.124, p < 0.001) and Shapiro–Wilk test (statistics = 0.940, p < 0.001). The histogram was right-skewed. Thus, the variable was transformed into categorical variable to define two levels of knowledge.

### Discussion

Parental awareness represents an essential component of the successful diagnostic and managemental approaches for children with amblyopia. In this study, the knowledge and awareness levels among parents and companions were assessed and showed a low-level knowledge, with only 25.9% of the participants having adequate knowledge based on our amblyopia knowledge scoring system, and 20% self-declaring adequate knowledge. The amblyopia definition was accurately perceived in only 36%. A recent study was performed to assess awareness about amblyopia among parents in Saudi Arabia [19]. Consistent with our data, they found that 30% of the participants were knowledgeable about the disease [19].

To the best of our knowledge, only one study has been conducted to assess amblyopia knowledge among the companions of children in Jeddah, Saudi Arabia [20]. Alzahrani et al. found that 49.7% of the participants were knowledgeable about the disease and its etiology, which is higher than our and Alsaqr et al.’s studies [19, 20]. This could be explained by the fact that their study sample included attendees of pediatrics and ophthalmology clinics at a tertiary hospital where there is more likely to be parents who are knowledgeable about the disease.

The level of awareness and knowledge regarding amblyopia in our study and other studies in Saudi Arabia are insufficient, but still they are markedly higher than some other countries such as India (3%) and Nigeria (2.9%) [19, 20].

In developed countries (Europe and North America), parental knowledge of eye diseases, including strabismus and amblyopia, as well as occlusion therapy was shown to be moderate [15, 16, 18].

In the current study, the main sources of knowledge were physicians and the internet, which is consistent with the study by Alsaqr el al. [19]. However, physicians are advised to provide information without the use of medical jargon [17, 21]. Additionally, it is important to deliver written information rather than verbal information because patients may forget or misinterpret up to 40% of the important instructions given by a physician [21].

Another important aspect of the physician–companion interaction is the language. This is highly concerning in Saudi Arabia because there are a large number of expatriate health workers. In this context, a recent systematic review showed that language differences represented a considerable barrier to provision of quality healthcare [22]. The effect of language difference was apparent in our study because Saudi parents were more knowledgeable about amblyopia compared to non-Saudi counterparts. Targeted awareness programs should consider providing information in a multilingual form to ensure a comprehensive delivery.

The internet contributed to consistently improving the participants’ knowledge in our study and recent studies about amblyopia [19, 20] and one of its main causes, strabismus [23]. Most parents/guardians (97.9%) used the internet to search for distinct information about their child’s health in a recent cross-sectional study in Canada [24]. Such information could be frequently obtained via public search engines which may lead to misinformation which would ultimately affect their attitudes. Therefore, maintaining reliable sources, such as governmental, hospital-based, and academic websites is important to provide correct information.

### Conclusion

Amblyopia is an easily treatable condition in children if the parents are aware and knowledgeable about the significance of early diagnosis and management. However, we showed that there was a lack of knowledge regarding different aspects of the disease, including basic information, causes, risk factors, and treatment.

## Limitations

- As with other questionnaire-based studies, the participants might misinterpret some questions.
- The role of physicians could not be interpreted because no data were available about the proportion of participants who attended ophthalmology clinics.

## Supplementary Information

**Additional file 1.** The questionnaire used in the study that was developed by the authors and underwent face and content validity.

**Additional file 2.** Factors associated with knowledge level about amblyopia (N=401).

## Data Availability

The data are not available for public access because of privacy concerns, but are available from the corresponding author on reasonable request.

## Abbreviations

SD: Standard deviation
Freq.: Frequency
