# Supplementary material for: Knowledge and attitudes regarding amblyopia among parents in Jeddah, Saudi Arabia: a cross-sectional study
Source: BMC Res Notes. 2021 Feb 10;14:60. doi: 10.1186/s13104-021-05478-y (PMC7877017; doi:10.1186/s13104-021-05478-y)
Supplement: Supplementary file 1 — Additional file 1. The questionnaire used in the study that was developed by the authors and underwent face and content validity. [file 13104_2021_5478_MOESM1_ESM.pdf]

This questionnaire aims to measure the community’s awareness about lazy eye disease (amblyopia). By filling out this questionnaire, you agree to use the information for scientific research purposes. All the information will be recorded anonymously. The data will be held only by the researches with strict confidentiality.

A : personal information :				
1	This data has been filled in by	○ The mother<br>○ The father<br>○ Other: (relationship: ……………………….)		
2	The Mother's educational level	Uneducated ☐ primary ☐ secondary☐ diploma /Institute ☐University ☐		
3	The Father's educational level	Uneducated ☐ primary ☐ secondary☐ diploma /Institute ☐University ☐		
4	Mother's Work	....	Age :	Nationality :
5	Father's Work	....	Age :	Nationality :

B: Knowledge-related questions		
6	Do you have adequate knowledge about amblyopia?	○ Yes<br>○ No
7	What is amblyopia?<br>**(choose only 1 answer)**	○ Decreased vision in one eye due to brain ignoring unclear image transmitted by the affected (lazy) eye, and favoring the healthy eye<br>○ A viral infection in the eye that causes progressive vision loss<br>○ Eye deviation (inwards or outwards) due to eye muscles imbalance.<br>○ Droopy eyelid (eyes appear sleepy)<br>○ I do not know .
8	What are the causes of amblyopia?<br>**(You can choose more than one answer)**	○ Squint (strabismus)<br>○ Congenital cataract<br>○ Droopy Eyelid<br>○ Corneal opacity<br>○ (premature babies)Premature birth<br>○ Myopia and farsightedness<br>○ Astigmatism<br>○ Genetic factors<br>○ Eye injuries (trauma)<br>○ I do not know
9	What are the symptoms of amblyopia?<br>**(You can choose more than one answer)**	○ Poor vision in one eye<br>○ Eye deviation (misalignment)<br>○ Abnormal head postures (such as head tilt or head turn)<br>○ Coming close to the television when watching, or bringing subjects close to the eye when looking at them<br>○ Headache or eye strain<br>○ I do not know
10	What are the risk factors for a child to develop amblyopia?<br>**(You can choose more than one answer)**	○ One of the parents has or had congenital cataract, droopy eyelids, strabismus, myopia, or hyperopia.<br>○ Family history (1st degree relatives) of congenital cataract or strabismus<br>○ (premature babies)Premature birth<br>○ I do notknow

11	What are the complications of not treating amblyopia? **(You can choose more than one answer)**	○ Permanent and irreversible vision loss in the affected eye.<br>○ Loss of 3 dimensional (3D) perception.<br>○ I do not know
12	What are the methods for treating amblyopia?<br><br>**(You can choose more than one answer)**	○ Laser therapy<br>○ Surgery<br>○ Cover the healthy eye and treat the underlying cause<br>○ Cover the affected (lazy) eye, and treat the underlying cause<br>○ Glasses only<br>○ Drops only<br>○ No treatment<br>○ I do not know
13	Does the child's age affect the response to treatment?	○ Yes<br>○ No<br>○ I don't know
14	Does amblyopia require lifelong treatment?	○ Yes<br>○ No<br>○ I don't know
15	Do you think amblyopia is a hereditary disease?	○ Yes it is. If no family members have amblyopia, then my kids will never get it.<br><br>○ No it is not. Any child can be affected by amblyopia.
16	Where do you get your information about amblyopia?	○ A doctor<br>○ Nurse<br>○ Medical Student<br>○ A family member<br>○ Internet<br>○ newspapers and magazines<br>○ the television<br>○ I don't have information<br>○ Others

**C: Attitudes questions**		
17	In your opinion, when is it necessary to take your child for an ophthalmology visit?	○ When the child complains (e.g blurry vision, double vision, eye pain)<br>○ When there is an abnormal sign (e.g strabismus or coming close to TV)<br>○ Periodic routine check-ups
18	Do you think amblyopia can be cured if the child complies with the treatment	○ Yes<br>○ No<br>○ I don't know
19	In your opinion, do parents have an essential role in the treatment of amblyopia?	○ Yes<br>○ No<br>○ I don't know

**D:**		
20	Do you have a child previously diagnosed with amblyopia?	○ Yes<br>○ No
