# Supplementary material for: Knowledge and attitudes regarding amblyopia among parents in Jeddah, Saudi Arabia: a cross-sectional study
Source: BMC Res Notes. 2021 Feb 10;14:60. doi: 10.1186/s13104-021-05478-y (PMC7877017; doi:10.1186/s13104-021-05478-y)
Supplement: Supplementary file 2 — Additional file 2. Factors associated with knowledge level about amblyopia (N=401). [file 13104_2021_5478_MOESM2_ESM.docx]

**Additional file 2:** Factors associated with knowledge level about amblyopia (N=401)

| **Factor** | **Category** | **Adequate** | | **Inadequate** | | **p-value** |
| --- | --- | --- | --- | --- | --- | --- |
|  |  | **Freq.** | **%** | **Freq.** | **%** |  |
| Guardian | Mother | 60 | 28.3 | 152 | 71.7 |  |
|  | Father | 35 | 22.6 | 120 | 77.4 |  |
|  | Other | 8 | 24.2 | 25 | 75.8 | .455 |
| **Mother’s factors** |  |  |  |  |  |  |
| Age | Mean, SD | 34.75 | 5.10 | 34.34 | 5.55 | .531 |
| Educational level | Low | 16 | 24.2 | 50 | 75.8 |  |
|  | High | 88 | 26.8 | 240 | 73.2 | .664 |
| Professional status | Housewife | 58 | 24.9 | 175 | 75.1 |  |
|  | Employed | 35 | 32.9 | 72 | 67.3 | .133 |
| Nationality | Saudi | 69 | 32.1 | 146 | 67.9 |  |
|  | Non-Saudi | 26 | 18.6 | 114 | 81.4 | .005* |
| **Father’s factors** |  |  |  |  |  |  |
| Age | Mean, SD | 41.33 | 6.56 | 40.45 | 6.63 | .283 |
| Educational level | Low | 13 | 28.3 | 33 | 71.7 |  |
|  | High | 91 | 25.9 | 260 | 74.1 | .735 |
| Nationality | Saudi | 66 | 31.1 | 146 | 68.9 |  |
|  | Non-Saudi | 27 | 18.6 | 118 | 81.4 | .008* |
| **Other factors** |  |  |  |  |  |  |
| Self-declared knowledge | No | 63 | 19.6 | 258 | 80.4 |  |
|  | Yes | 41 | 51.3 | 39 | 48.8 | <.001* |
| Having a child with amblyopia | No | 86 | 25.1 | 257 | 74.9 |  |
|  | Yes | 18 | 31.0 | 40 | 69.0 | .338 |

Because of missing data, some values do not add up to the total.

Freq, frequency; SD, standard deviation; *Statistically significant result (*P* < 0.05).
